# Expression classifiers for developmental toxicants

**DOI:** 10.17179/excli2015-765

**Published:** 2015-12-22

**Authors:** Raymond Reif

**Affiliations:** 1 Leibniz Research Centre for Working Environment and Human Factors at TU Dortmund (IfADo), Ardeystrasse 67, 44139 Dortmund, Germany

## ⁯⁯

In its recent edition the Archives of Toxicology have published a transcriptome-based classifier that identifies developmental toxicants acting as histone deacetylase inhibitors. The authors, Eugen Rempel and colleagues from Dortmund Technical University, demonstrate that based on only eight genes the gene expression pattern of developmental toxicants acting as histone deacetylase inhibitors can be identified. They used a test system that recapitulates generation of neuroectoderm from pluripotent stem cells (Balmer et al., 2014[1]; Zimmer et al., 2014[27]; Leist et al., 2013[14]; Stöber et al., 2014[23]; Reif, 2014[19]). Exposure of the test cells for six days to six histone deacetylase inhibitors (HDACi) caused alterations in expression profiles that could be clearly differentiated from other compound classes, such as a heterogeneous group of 'mercurials'.

Currently, classification and grouping represents a cutting-edge topic in toxicological research (Gocht et al., 2015[5]; Godoy et al., 2015[7], 2013[6]; Grinberg et al., 2014[8]; Shinde et al., 2015[21]; Meganathan et al., 2015[16]; Weng et al., 2014[25]; Krause et al., 2013[10]; Gebel et al., 2014[4]). To reach this goal in the field of developmental toxicity, stem cell based test systems have been developed that recapitulate different phases of human development (Krug et al., 2013[11]; Leist et al., 2013[14]; Bolt, 2013[2]; Hoelting et al., 2013[9]). Using these systems it has been shown that three concentration ranges can be differentiated, namely tolerated concentrations where no gene expression changes occur, the teratogenic range which leads to deregulation of critical developmental genes and the cytotoxic concentration range where additional genes associated with cell death and catabolic metabolism are observed (Waldmann et al., 2014[24]). Importantly, the 'teratogenic concentration range' *in vitro* overlapped with concentrations known to cause teratogenic effects in humans *in vivo*. A next challenge in test system development is to develop expression signatures which indicate that specific toxic mechanisms are active. In this respect the present work of Rempel et al., using a set of structurally not related compounds acting by a similar mechanism, represents an important proof of concept (Rempel et al., 2015[20]). Next open questions to be addressed are whether the test systems correctly identify further classes of developmental compounds and differentiate them from compounds acting by unspecific toxic mechanisms or substances that preferentially cause other types of toxicities. In the past and also presently developmental toxicity has mostly been tested *in vivo* (Lee et al., 2011[13], 2007[12]; Liu et al., 2010[15]; Moss et al., 2009[17]; Xi et al., 2009[26]; Oesch et al., 2008[18]; Stapleton and Chan, 2009[22]; Ejaz and Woong, 2006[3]). Although some first important steps have been achieved in this field of in vitro research there seems to be a long way to go until animal experiments in developmental toxicity can be fully replaced by alternative methods.
